# Negative pressure pulmonary oedema and haemorrhage, after a single breath-hold: Diaphragm the culprit?

**DOI:** 10.4103/0019-5049.68391

**Published:** 2010

**Authors:** John George Karippacheril, Tim Thomas Joseph

**Affiliations:** Department of Anaesthesiology, Kasturba Medical College, Manipal, Karnataka - 576 104, India

Sir,

A 26-year-old, moderately built male, ASA PS1, no history of snoring, respiratory infection, bronchial asthma or allergies, with osteoblastic lesions of distal femur and L4-5 vertebrae, underwent closed vertebral biopsy in prone position under general anaesthesia.

Anaesthesia was maintained with isoflurane, nitrous oxide and oxygen. At the end of procedure, adequate neuromuscular reversal was achieved, as assessed by double burst stimulation on peripheral nerve monitor. An awake extubation was planned; however, on return of protective airway reflexes, patient started persistently coughing and bucking on the endotracheal tube. Through an oral airway, thorough suctioning was done prior to extubation. An episode of apparent breath-holding (held in inspiration, absent chest movements, with no capnographic trace after a downstroke, no audible stridor), lasting less than a minute after noticing it, was observed. Face mask ventilation was immediately initiated with difficulty. SpO_2_ suddenly dropped to 51% and heart rate increased to 108/min. Continuous Positive Airway Pressure (CPAP) with 100% O_2_ was immediately given with APL valve closed. SpO_2_ returned to 98% within a few seconds. Auscultation revealed bilateral fine crepitations. Ten milligram of IV frusemide was given on suspicion of negative pressure pulmonary oedema, IV dexamethasone 8 mg was also given.

Intraoperatively, the patient received 1500 mL crystalloid, there was no major blood loss. Patient was shifted to post-operative care unit as SpO_2_ was stable at 98% on 6L/min O_2_ via facemask. Immediate chest X-ray was asked for. Thirty minutes after the episode, in the post-operative area, his room air saturation showed 77 to 81%. He was tachypneic (respiratory rate about 28 per minute) but not distressed, coarse crepitations were present bilaterally on auscultation, with expectoration of copious quantity of serosanguineous frothy sputum, progressing to several episodes of more sanguinous expectoration.

**Figure 1:**
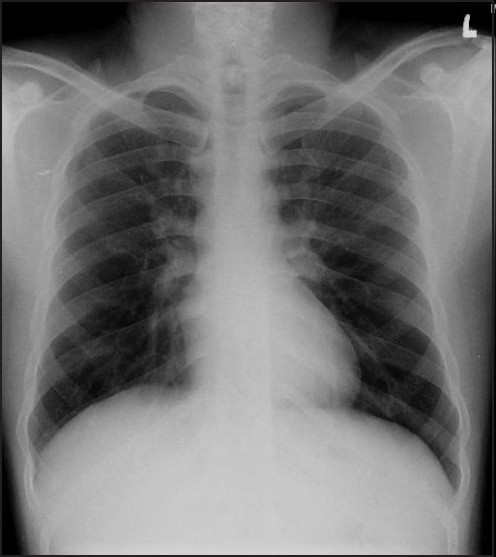
Pre-operative normal chest X-ray

**Figure 2:**
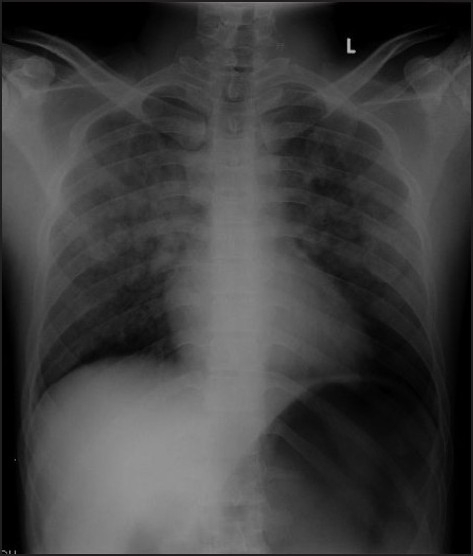
Thirty-minute post-operative chest X-ray, showing diffuse infiltrates more in mid-zone perihilar region, obvious air shadow in gastric fundus

Chest X-ray showed significant diffuse especially perihilar infiltrates, suggestive of pulmonary oedema and alveolar haemorrhage, with prominent gastric air shadow compared with a normal pre-operative chest X-ray [Figure 1 and 2]. ABG done on oxygen with 60% Venturi by face mask showed PaO_2_ 63.7 mmHg, pH 7.45, PaCO_2_ 29.7 mmHg, HCO_3_ 20.4 mmol, Base excess (BE)-2.2. ICU care and non-invasive ventilation with 8 cm H_2_O PEEP and diuresis with IV frusemide 20 mg bolus was started. Ryle’s tube was inserted to decompress the stomach.

**Figure 3:**
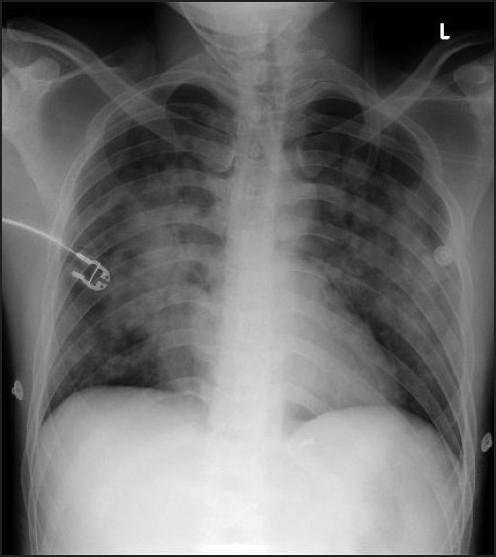
Second post-operative day chest X-ray, showing prominent mid-zone perihilar infiltrates

**Figure 4:**
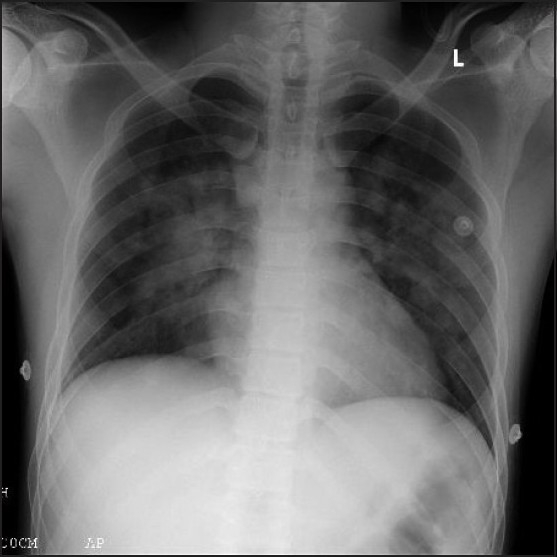
Third post- operative day chest X-ray, showing resolving mid-zone perihilar infiltrates.

Frothy expectoration reduced within few hours, ABG showed improvement of PaO_2_ to 232 mmHg on FIO_2_ 0.6, pH 7.47, PaCO_2_ 31.8 mmHg, HCO_3_ 22.7 mmol/L, BE 0.2. In 72 h, the patient was discharged with stable parameters, maintaining PaO_2_ of 105 to 106 mmHg on nasal prongs with 2 L/min O_2_ flow. Radiological features suggestive of alveolar haemorrhage persisted over the next few days [Figure3 and 4], even after clinical resolution of symptoms; room air SpO_2_ was 98% on third post-operative day.

Unrecognized negative pressure pulmonary oedema (NPPE) is associated with a higher morbidity and mortality, with progression to acute respiratory distress syndrome, hypoxemia and death.[1]

In our case, we immediately appreciated fine crepitations over both sides of chest, which gave an early clue to the possibility of NPPE. The apparent breath-holding may have been a forceful sustained inspiration, produced by a powerful diaphragmatic contraction, held against a closed glottis (Muller’s manoeuvre), which can generate negative inspiratory pressures of up to -100 cm H_2_O.[2] Normal inspiratory efforts may not be sufficient to generate such an excessive negative pressure; diaphragmatic contractions may be the main contributing factor.[3] Whether subtle diaphragmatic contractions occurred during breath-holding, contributing to NPPE is not known.

As previously reported in literature, our patient had clinical resolution of symptoms despite radiological persistence of perihilar infiltrates even after three days, suggestive of slow pathological resolution of alveolar haemorrhage or interstitial oedema.[4,5]
